# A New Perspective on Assessing Cognition in Children through Estimating Shared Intentionality

**DOI:** 10.3390/jintelligence10020021

**Published:** 2022-03-29

**Authors:** Igor Val Danilov, Sandra Mihailova

**Affiliations:** 1 Academic Center for Coherent Intelligence, LV-1050 Riga, Latvia; 2 Faculty of Communication, Rīga Stradiņš University, LV-1007 Riga, Latvia; sandra.mihailova@rsu.lv

**Keywords:** intelligence, computerized diagnostic assessment, shared intentionality, interpersonal dynamics

## Abstract

This theoretical article aims to create a conceptual framework for future research on digital methods for assessing cognition in children through estimating shared intentionality, different from assessing through behavioral markers. It shows the new assessing paradigm based directly on the evaluation of parent-child interaction exchanges (protoconversation), allowing early monitoring of children’s developmental trajectories. This literature analysis attempts to understand how cognition is related to emotions in interpersonal dynamics and whether assessing these dynamics shows cognitive abilities in children. The first part discusses infants’ unexpected achievements, observing the literature about children’s development. The analysis supposes that due to the caregiver’s help under emotional arousal, newborns’ intentionality could appear even before it is possible for children’s intention to occur. The emotional bond evokes intentionality in neonates. Therefore, they can manifest unexpected achievements while performing them with caregivers. This outcome shows an appearance of protoconversation in adult-children dyads through shared intentionality. The article presents experimental data of other studies that extend our knowledge about human cognition by showing an increase of coordinated neuronal activities and the acquisition of new knowledge by subjects in the absence of sensory cues. This highlights the contribution of interpersonal interaction to gain cognition, discussed already by Vygotsky. The current theoretical study hypothesizes that if shared intentionality promotes cognition from the onset, this interaction modality can also facilitate cognition in older children. Therefore in the second step, the current article analyzes empirical data of recent studies that reported meaningful interaction in mother-infant dyads without sensory cues. It discusses whether an unbiased digital assessment of the interaction ability of children is possible before the age when the typical developmental trajectory implies verbal communication. The article develops knowledge for a digital assessment that can measure the extent of children’s ability to acquire knowledge through protoconversation. This specific assessment can signalize the lack of communication ability in children even when the typical trajectory of peers’ development does not imply verbal communication.

## 1. Introduction

This extended communication article presents groundbreaking preliminary results and significant findings from our more extensive study over multiple years (Communication article types of MDPI journals), complementing them with relevant studies of other researchers already presented in the literature. This theoretical manuscript aims to create a conceptual framework for future research on digital methods for assessing cognition in children through estimating shared intentionality, different from assessing through behavioral markers. The core idea of this digital method for assessing cognition in young children is the evaluation of an extent of shared intentionality (ShI) in dyads made by analyzing their protoconversation. This interaction modality occurs in child-adult interaction at the onset of cognition, during the period of development when children still lack communication skills (Gopnik 1981; Trevarthen 1989, 1998; Yingling 1990; Bråten et al. 1998; Tomasello 2019). It appears before the age when the typical developmental trajectory predetermines communication. Even though the protoconversation succeeds through emotion sharing (Tomasello 2019), this mechanism is unclear (Danilov and Mihailova 2021c). In newborns, emotion sharing can only proceed without sensory cues. Indeed, neonates can not perceive emotion expressions of others due to a lack of social knowledge and reduced perception. It is not clear how newborns could be aware enough of their movements and how these map onto the movements of others (Jones 2009, 2016). Therefore, a hypothesis of inborn emotion universalism (Ekman 1999) cannot advocate the protoconversation through sensory cues. Moreover, this universality hypothesis is formed by limited experimental methods since other research designs show the opposite outcome (Jack 2013; Crivelli and Gendron 2017; Barrett et al. 2019; Hoemann et al. 2019; Siegel et al. 2020). Empirical evidence shows that emotion sharing happens without sensory cues and even awareness of the existence of emotional stimuli (Tamietto et al. 2009). Social interaction without sensory cues has been recently registered and presented in the neuroscience literature, showing coordinated neuronal activities in subjects in the absence of sensory cues between them (e.g., Painter et al. 2021; Fishburn et al. 2018; Valencia and Froese 2020). Recent studies also show an increase in acquiring new knowledge in the same conditions for subjects (Danilov et al. 2019; Danilov and Mihailova 2020, 2021a, 2021b). However, the studies on interaction in the mother-child dyads without sensory cues are very limited (e.g., Danilov et al. 2021a, 2021b).

The article is outlined as follows. The introduction observes our knowledge about interpersonal dynamics that contributes to predicting social reality. The empirical evidence shows that neurobiological harmony promotes shared intentionality through step-by-step ongoing coordination of cyclically enhanced movements under ever-growing emotional arousal. Subsequently, it illustrates the current knowledge on a contribution of shared intentionality to cognition. In Section 2 the research problem is explained; what experiments showed incredible newborns’ achievements and were conducted under emotional stimulation even in a case when experimenters did not take this condition into account? Is an unbiased assessment of the interaction ability of children is possible before the age when the typical developmental trajectory predetermines verbal communication? Then, in Section 3 the method of selecting articles for two steps of the research is presented. The analysis of experimental data establishes Section 4. It consists of two parts. Firstly, this data selection allows the observation of shared intentionality in different experimental modes; and secondly, evaluation of whether the extent of shared intentionality is measurable. Section 5 discusses the results, as well as the limitations of this new approach to assessing cognition in children and the limitations of the current study. It also presents an implication of this study for indicating development trajectory in children. Finally, Section 6 concludes the paper by summarizing the contents.

### 1.1. Triggers of Shared Intentionality

Survival in a changing environment requires qualities that predict social reality (Prochazkova and Kret 2017; Adolphs 2001). Our reasoning looks for patterns of information that provide predictions. One efficient way to predict social reality is the temporal coordination of movements with co-mates. The brain tries to make it easier for itself to predict others’ behavior without communication since coordinated repetitive motions of intimately related individuals are more predictable ones for the brain. Cognitive development and social cognition require individuals to link with relatives (and/or co-mates) and coordinate with them in order to capture patterns of information that provide predictions. Indeed, empirical evidence shows that our perception looks for synchrony in external motions (Dobbins and Grossmann 2018). Growing evidence shows capturing of social cues in coordinated interaction in mothers and infants through unintentional mirroring: social entrainment (e.g., Aschoff 1963; Grandin et al. 2006), early imitation (e.g., Meltzoff and Moore 1977; Meltzoff 2005) and interactional synchrony (e.g., Condon and Ogston 1967; Markova et al. 2019). Research highlights that emotional sharing increases infants’ mirroring actions (Anisfeld 2005; Jones 2009; Vincini et al. 2017). The impact of emotional arousal on group performance is well studied (e.g., Hebb 1955; Miller et al. 2007; Cirelli et al. 2020).

**Figure 1:**
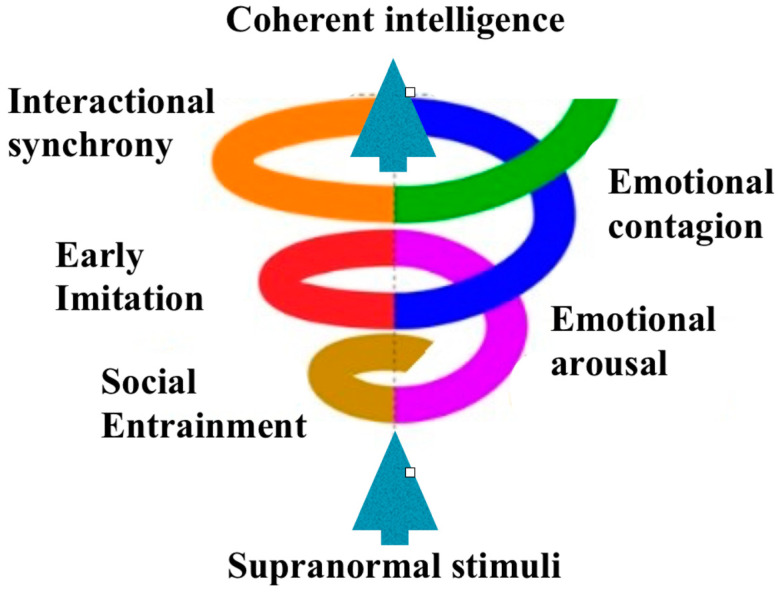
The Model of Coherent Intelligence in interpersonal dynamics of individuals from psychophysiological coherence to coherent intelligence (Reprinted with permission from Danilov and Mihailova 2020).

According to Danilov and Mihailova (2020), the Model of Coherent Intelligence defines interpersonal dynamics that create shared intentionality in the mother-child dyads (see Figure 1, Danilov and Mihailova 2020). Interpersonal dynamics appear in a supranormal environment (e.g., first hours after birth). This particular environment stimulates supranormal sensation in mother-child dyads. At the same time, the inherited mechanism of infants’ social entrainment to the mother’s rhythm is activated. Both the supranormal sensation and social entrainment may stimulate common emotional arousal. The latter is increased by the ongoing supranormal sensation and the occurring rhythm of arbitrary movements of the newborn. The ever-increasing arousal in the dyad and the rhythm of the neonate’s unintentional movements stimulate emotional contagion. Interactional synchrony becomes the zenith of these interpersonal dynamics, coming through common emotional arousal with the identical rhythm of movements (imitation). In this way, neurobiological harmony promotes shared intentionality through step-by-step ongoing coordination of cyclically enhanced movements under ever-growing emotional arousal. This model defines the main extrinsic characteristic of shared intentionality manifestation (probably, the only extrinsic one): emotional arousal. Social entrainment is perhaps the only intrinsic property that distinguishes groups with the “shared intentionality” quality from other groups without it.

### 1.2. Shared Intentionality Contributes to Cognition

According to Tomasello (2019), social bond development in children refers to time slices: (1) emotion sharing from birth, (2) joint intentionality from the nine-month revolution, (3) collective intentionality at around three years of age, (4) reason and responsibility. Tomasello (2019) introduced the beginning of cognition through the newborns’ primary motive force of Sharing Intentionality (ShI). ShI is an essential tool for protoconversation that appears in the lack of communicative abilities, i.e., without interaction with symbols. A growing body of literature shows neuronal synchronization that appeared across brains during meaningful social interaction (Oldham et al. 2018; Valencia and Froese 2020). Recent findings extend knowledge about human cognition and interpersonal interaction by showing an increase of both neuronal activities (Painter et al. 2021) and acquisition of new knowledge (Danilov et al. 2019; Danilov and Mihailova 2021a, 2021b) in subjects in the absence of sensory cues between them (any verbal and non-verbal communication) in all the above experiments. These outcomes highlight the contribution of interpersonal interaction to cognition from the onset, discussed already by Vygotsky (2016). This coordinated neuronal activity during meaningful social interaction without sensory cues indicates a coherent mental process that shapes ShI in intimately related individuals in social entrainment (Danilov and Mihailova 2021b). ShI promotes the first step in protoconversation in mother-child dyads (Tomasello 2019). In such a group with interpersonal dynamics, individuals can instantly select only one stimulus for the whole group (from a noisy environment with many irrelevant stimuli), even without communication between them (Danilov and Mihailova 2021b, 2021c). In dyads, ShI creates links between sensory cues and social phenomena (familiar to mature organisms) in young children, facilitating their categorization of reality—understanding phenomena during learning (Danilov et al. 2021a). According to Danilov et al. (2021b), ShI contributes to a subjectively distinct perception of sudden and unexpected insights when solving problems in the learning process. This outcome is compatible with the concept of Coherent Intelligence (CI)—the result of the mental coherence of individuals due to ShI (Danilov and Mihailova 2021c).

## 2. Research Problem: How Is Cognition Related to Emotions in Interpersonal Dynamics?

The manuscript is creating a conceptual framework for future research to study whether a digital assessment of the interaction ability of children is a possible tool for assessing their cognitive development before the age when the typical developmental trajectory implies verbal communication. We divide the research problem of this study into two steps. Firstly, we discuss whether previous research already registered that protoconversation without sensory cues can promote cognition in children. Therefore, we concern ourselves with studies of other researchers showing the impact of emotional arousal on intellectual performance in mother-child dyads. At this step, the manuscript determines studies that showed incredible newborns’ achievements; we are assuming emotions as independent variables even though experimenters did not consider them. Secondly, we study whether reduced interaction modality (reduced protoconversation) detects a developmental delay in children even if it is measured online before the age when the typical developmental trajectory implies verbal communication, as well as whether or not the extent of protoconversation is measurable. This Section clarifies the above-noted research questions.

If emotions influence cognition from the very beginning, then the empirical data on child development should show numerous results of this influence. If protoconversation shapes cognition, then the vast body of literature should contain facts about the manifestation of shared intentionality. The above-noted literature shows that protoconversation happens without sensory cues and even awareness of subjects about such a mode of social interaction. Therefore, the first research question is whether emotions were independent variables in different research designs on child behavior even though experimenters did not consider this condition. We suppose that while experimenters did not consider that emotion could directly affect the dependent variable, emotional stimulation was the independent variable in these experiments. For this reason, Section 4 compiles outcomes of experiments that showed incredible achievements of newborns and were conducted under emotional stimulation even when experimenters did not consider this condition. This will show that newborns’ intentionality in these experiments could appear even before it is possible for children’s intention to occur. We believe this could happen due to the caregiver’s help under emotional arousal. The emotional bond evokes intentionality in neonates. Therefore, they can manifest unexpected achievements while performing them with caregivers.

The current study discusses a new approach to the early diagnosis of developmental delay by measuring social interaction in caregiver-child dyads. It is a new assessment mode for earlier detection of developmental delays, different from assessing through behavioral markers, which are not considered protoconversation, instead relying on interaction via sensory cues only. Children’s lack of interaction ability indicates a developmental delay (Erikson 1963; Winnicott 1973; Gresham and McMillan 1997; Cullinan 2002; Prior and Ozonoff 2007; Sternberg and Sternberg 2012; Vygotsky 2016; Pierce et al. 2019; Rojas-Torres et al. 2020). For instance, specific deficits of infantile autism syndrome appear in imitation, emotion sharing, theory of mind, the pragmatics of communication, and symbolic play (Rogers and Pennington 1991). Computerized testing could objectively assess infants’ interaction abilities even at distance if it measures the extent of their cooperation with caregivers in problem-solving without communication. For this reason, the research aims to create a concept design to develop a digital assessment tool to detect online a developmental delay in children at the earliest stage. Defining the research problem, the article discusses whether the extent of protoconversation is measurable. In other words, the second research question is whether an unbiased digital assessment of the interaction ability of children is possible before the age when the typical developmental trajectory implies verbal communication. This discussion can develop a framework for future research on a digital method for assessing cognition in young children.

## 3. Materials and Methods

Due to the twofold research problem, the analysis also proceeds with two steps. In the first step, we distinguish two sorties of studies on young children’s behavior: (i) the articles that show infant behavior consistent with generally accepted knowledge of child development; and (ii) those that demonstrate unexpected children’s achievements. The unexpected achievements are surprising outcomes that show more developed children’s behavior than those are expected by generally accepted knowledge on child development. Then, we compare their outcomes to reveal conditions that differ between these two modes. A growing body of experimental data shows that newborns are able to categorize different social cues (unfamiliar for them), e.g., the other-race effect (Pascalis and de Schonen 1994; Kelly et al. 2007), and recognizing faces by their parts (Simion et al. 2007). Even though their research design did not control and even consider this interaction in dyads, these experiments could manifest outcomes of protoconversation of young children with caregivers. Section 4.1 discusses this issue.

In the second step, we collected the studies to analyze their empirical data and determine if they reported experimental results on efficient decision-making in mother-infant dyads without sensory cues, comparing the performance of young children with a developmental delay with their peers. However, the studies with the intellectual outcome (with the outcome, that means the acquisition of social knowledge in children) in protoconversation without sensory cues are very limited. Moreover, there are no data for comparing such performance in children of different cognitive developments. Recent studies (Danilov et al. 2021a, 2021b) obtained this result. In such experiments, they compared the performance of young children with a developmental delay with their peers. While the authors of the chosen papers have already presented these results at a conference (Danilov et al. 2021a, 2021b), this is the first interpretation of the data from the viewpoint of an assessment of development trajectories in infants. 

Our recent case study (Danilov et al. 2021a) observed subjects’ ability to associate the sound of spoken numbers from one to three (in an unfamiliar language) with the corresponding set of items. The research method checked the extent of protoconversation in dyads by eliminating any communication between them via sensory cues—ShI efficiency. This study hypothesized that if shared intentionality promotes cognition from the onset, this interaction modality can also facilitate insight into numeracy in older children. According to the received view, numerical competence develops in children at the toddler stage of development. Children reach the meaning of numeracy after the age of about 36 months. At the age of 18 months, most children still do not speak. Non-native language for instructions during testing also makes testing harder for children. That is, this online study (Danilov et al. 2021a) observed pure protoconversation in the dyads by testing children in an unfamiliar language without counting skills. Section 4.2 discusses the results of this study. Briefly, the research method is outlined as follows.

Subjects. Five children participated in the experiment. Children within the normal developmental trajectory aged 12 months, 18 months, 28 months, and 31 months were invited with their mothers for the online research as the control group. The girl aged 33 months with a developmental delay was tested with her caregiver.

Stimuli. The stimuli were sets of balls (from one to three in each set), presented on the screen for 30 s in each trial. 

The tasks. Children were asked to show the set with the correct number of items by selecting one among three options on a mobile phone screen. It is noteworthy that the child needed, firstly, to sort out the question: what the experimenter was asking to do and how many balls he needed. Secondly, the child had to find the right combination with the appropriate number of balls. The children’s task was to choose the set of balls that the experimenter asked in an unfamiliar language (English for Italian and Russian native speaking dyads, the caregivers understood these instructions in English).

Materials. The training program provided a series of 10 different numeric tasks. The answers were presented to subjects via specific software on a mobile device. This software also induced interactional synchrony between the subjects and their mothers. The rhythmically changing red/purple colors of mobile phone screens caused emotional arousal. Moreover, just the participation in such an unusual performance as an experiment excited the subjects. Unintelligible tasks also stimulated emotional arousal in the subjects, as well as the fact that a stranger-teacher (the experimenter) participated in the performances. The software produced rhythmically changing colors of the mobile phone screen (80 beats per minute) to stimulate interactional synchrony in the dyads. According to the model of coherent intelligence (Danilov and Mihailova 2020), emotional contagion and interactional synchrony provide shared intentionality in dyads. In such a manner, human-computer interaction stimulated shared intentionality for the child’s choice of the correct answer from three options on the smartphone screen. 

Procedure. The experiments were conducted online via video conversation. The dyads were seated behind the computer (videoconference regime) with one smartphone at a distance of 0.4 m. from their eyes. During the session, the children independently performed the test on the mobile phone, which the caregivers held in their hands. Any communication in the dyads was excluded. In each trial, the children were asked: “please show me (number from one to three of) balls”. The experimenter asked this phrase in the children’s native language (Italian or Russian) except for the number of balls pronounced in English.

Evaluation of protoconversation. The experimenters expected that if this computer testing could measure the interaction ability in infants, then the reduced intellectual outcome in testing could mean reduced protoconversation (comparing with normal trajectory baseline). Infants could not correctly answer on unintelligible and unfamiliar tasks independently. Therefore, their decreased performance could mean reduced protoconversation and possible developmental delay due to socio and/or biological causes.

## 4. Results: Detecting Shared Intentionality to a New Assessment Mode

This section interprets the results of the chosen studies for reasoning that emotion-motion interpersonal dynamics shape shared intentionality. We show that the emotional bond evokes intentionality in neonates. Thanks to this emotional contagion, newborns can successfully solve unfamiliar (and even unintelligible) tasks by performing them with caregivers. This literature analysis of the twofold task consists of two parts. Firstly in Section 4.1, the data selection allows observation of shared intentionality in two different experimental modes. We discuss the impact of interpersonal dynamics (in specific, emotional arousal) on intellectual performance by distinguishing two types of studies on young children’s behavior. Secondly, in Section 4.2, the current study hypothesizes that if shared intentionality promotes cognition from the onset, this interaction modality can also facilitate cognition in older children. Therefore, it analyzes recent studies with the question: how can the extent of shared intentionality be measured in children aged 18 months, 28 months, 31 months, and 33 months?

### 4.1. Two Modes of Research with/without Emotional Arousal 

The current paper distinguishes two experimental conditions in detecting intentions in infants from the literature on children’s development: “Observer” and “Experimenter”. Studies within the “Observer” paradigm simply inspect infants’ behavior in daily routine without introducing special conditions for inducing subjects’ reactions during observation. Studies within the “Experimenter” paradigm mainly create specific conditions (usually unfamiliar and unexpected phenomena) for the subjects to stimulate their reactions. 

Specifically, the “Observer” research design studies self-directed behavior, verbal labeling behavior, and differential responding to different stimulus conditions of daily routine; all might indicate self-recognition (Brooks-Gunn and Lewis 1984). It seems uncontroversial to say that self-recognition in infants manifests their intention–their ability to manifest intention towards themselves. Nevertheless, at what point in the ontogenetic sequence does self-recognition occur? Brooks-Gunn and Lewis (1984) highlighted six different behavioral scripts that could be attributed to self-recognition at an early age:

(i) An infant seems not to have looked at her hands before the age of 2 (30)–two months (thirty days).
(ii) At age 3(22) months, an infant’s glance follows her hands. Hands turn aside, and she seems very much surprised to see them reappear.
(iii) An infant at age 2(8) months always pulls at her face before, during, or after sucking his fingers.
(iv) An infant at age 2(17) months babbles and smiles without any desire to suck while holding his nose with his right hand.
(v) An infant at age 4(15) months repeatedly smiled at the caregiver’s image and his own in a mirror and no doubt mistook them for real objects.
(vi) A 9-month-old associated his name with his image in the looking-glass.

The literature shows that in a natural environment (without emotional arousal due to experiment), newborns do not play an active role in interaction with the mother (e.g., Lavelli and Fogel 2002; Kugiumutzakis 1999; Trevarthen 1998; Reddy et al. 1997). These considerations may mean that intention appears in infants no earlier than 2 months of age. The above points are also consistent with Piaget’s theory of cognitive development. After Piaget, we know that during the first reflexes substage, an infant only manifests pure reflexes–intentional actions emerge in infants no earlier than 2 months of age. Piaget (1963) argued that children’s actions are simply biologically given action patterns in the earliest stages of development. Moreover, actions can be goal-directed but not intentional because the internal goals of the infant can only be activated by the immediate stimulus environment (Piaget 1963). Such behavior is internally activated when the child can create goals and plans in the absence of external events directly relevant to those goals and plans (Piaget 1963). The goal-directed behavior is pure reflexes because the “goal” of an action is the action itself; the action is not differentiated from its goal as a means to an end (Chapman 1990). Piaget’s theory has been criticized and amended: the stages tend to overlap, and the boundaries are fuzzier than Piaget claimed (Borgers et al. 2000). However, children’s cognitive capacities do clearly increase with age; the Piaget’s stages should not be seen as sharply distinct categories, but rather as a moving scale (Borgers et al. 2000). In any way, Piaget’s developmental stages are a general framework for numerous researchers of child development (e.g., Borgers et al. 2000; Huitt and Hummel 2003; Carden 2018; MacNeill et al. 2018; Carpendale et al. 2019; Nagai 2019). The findings within the “Observer” paradigm are also consistent with many parents’ observations of their children. Many readers are parents themselves and may support these conclusions with their own experience.

On the contrary, research within the “Experimenter” paradigm shows extremely intriguing results. Neonates manifested numerous social actions in the first days and even the first hours of their life (Danilov 2020). A growing body of research show evidence of such newborns’ achievements as a reaction to crying of another newborn (Geangu et al. 2010; Dondi et al. 1999; Martin and Clark 1982; Sagi and Hoffman 1976; Simner 1971), early imitation (e.g., Meltzoff and Moore 1977; Meltzoff 2005; Nagy et al. 2013; Simpson et al. 2014), other-race effect (e.g., Pascalis and de Schonen 1994; Kelly et al. 2007), recognizing faces by their parts (Simion et al. 2007), recognizing faces without their context in schematic pictures (Goren et al. 1975; Johnson et al. 1991), facial attractiveness (Quinn et al. 2008), distinguishing mother and stranger (e.g., Bushnell et al. 1989; Bushnell 2001; Field et al. 1984; Pascalis et al. 1995), other-species effect, and others (Danilov 2020). Research under the “Experimenter” paradigm detect newborns’ actions, which seem intentional from birth.

### 4.2. Towards a New Assessment Paradigm

Can the extent of shared intentionality be measured in children? Danilov et al. (2021a) reported experimental data of ShI efficiency in children aged 18 months, 28 months, 31 months, and 33 months. These case studies tested ShI in dyads with young children of the standard developmental trajectory (18 months, 28 months, 31 months of age), comparing their results with a child with a developmental delay (aged 33 months). The 7 min, online computer-based assessment testing verified children’s ability to acquire knowledge through ShI in the absence of sensory cues. The experimental design checked their ability to interact with mothers without communication between them by asking dyads to create the bond between sounds of spoken numbers (in unfamiliar language for children while familiar for their mothers) and the appropriate set of items (Danilov et al. 2021a). Children aged 28 months and 31 months, and even the non-speaking child of 18 months old, showed significant interaction with mothers by presenting a ratio of correct responses above chance; respectively, 114%, 87%, and 125%. However, the child with developmental delay showed three times lower results on numerical competence–33%. 

Two case studies with 28-month-old and 33-month-old children (18 and 12 lessons of 20 min each, respectively) show that young children can predictably capture the meaning of numeracy during the short online course (Danilov et al. 2021b). Children have become “cardinal principle” knowers at an unexpectedly young age when other children usually do not comprehend numeracy (Danilov et al. 2021b). At the same time, it is generally argued across research that children reach understanding numeracy after the age of about 36 months; and this unpredictable process can last until the fifth year of age (Wynn 1992; Feigenson et al. 2004; Izard et al. 2009; Coolidge and Overmann 2012; Gärdenfors and Quinon 2020; Schneider and Barner 2020).

## 5. Discussion

Two experimental conditions in detecting intentions in infants—”Observer” and “Experimenter”—were distinguished from the literature on children’s development. The data show that in the “Observer” paradigm, the intention appears in infants no earlier than 2 months of age. The generally accepted theory of cognitive development (supported by decades of observation of children’s behavior) and our knowledge from everyday life also indicate that children’s performance within the “Observer” paradigm shows an intention not earlier than 2 months of age. On the contrary, research under the “Experimenter” paradigm detects newborns’ actions which seem intentional from birth. Therefore, these results show a dichotomy; research under “Observer” and “Experimenter” paradigms contradict each other. 

Furthermore, the results under the “Experimenter” paradigm also contradict our knowledge about infants’ abilities. The facts from human anatomy and children’s development doubt the neonates’ achievements under the “Experimenter” paradigm because young children could not perform these actions on their own (the facts below support this); they could not do them independently (alone). Newborns’ perception of reality is limited due to physiological reasons. The graduate development of children’s eyes is well known. Neonates and infants can not perceive the same picture of reality as adults possess. Their picture is significantly reduced because of immature (underdeveloped) eyes. According to Dobson and Teller (1978), an infant is able to focus on a clear image on the retina, while the fovea and other visual parts of the brain are too immature to transmit a clear image. Visual acuity in a neonate is 12 to 25 times worse than in an average adult (Slater 1995; Kellman and Banks 1998; Skoczenski and Norcia 2002). The cones of newborns are much less sensitive to light than those of an adult—by some estimates, by a difference of 350 to 1 (Kellman and Banks 1998). Although newborns might not be color-blind, they apparently do not perceive much in the way of color. When differences in brightness are controlled, infants fail to discriminate among a wide range of colors until about 8 weeks of age (Allen et al. 1988). Research has shown that newborns can likely discriminate between the colors red and white but cannot differentiate blue, green, and yellow from white (Adams et al. 1994). Newborns’ hearing is a bit worst then adults’ (Trehub and Schellenberg 1995). To hear a sound clearly, newborns require that sound to be about 15 decibels louder than adults need. For instance, a typical conversation is about 60 decibels. 

Sensing simultaneously maintains a massive number of stimuli. For instance, sensing is full of visual stimuli that perceiving may detect in the range of 400–790 THz. Sounds from the range of 20 to 20,000 Hz may simultaneously stimulate hearing. Many stimuli pressure the human body, exciting tactile sensing. Proprioception, olfaction, and even thermal stimuli enrich the chaos of stimuli. Each instant, this cohort of different stimuli simultaneously affects the nervous system through receptors. Our reasoning (mind) connects stimuli to symbols that have meanings. At the onset of cognition, the reduced sensing leads to reduced perception of objective reality–newborns’ reality should be different due to the above-noted physiological reasons. For this reason, if even categorizing objective reality in the same way as adults do is a difficult task for newborns, social reality is a much more complex problem for them.

Limited perception and lack of social knowledge in newborns do not allow them to recognize social symbols on their own. Two research paradigms yield incredibly different outcomes. From the “Observer” paradigm perspective, infants’ intention seems to appear no earlier than 2 months of age. In contrast, the experiments under the “Experimenter” paradigm show more significant newborns’ achievements, implying their intentionality towards phenomena. Even the newly born neonates under the “Experimenter” paradigm could solve tasks that were inaccessible for young children under the “Observer” paradigm. Under the “Experimenter” paradigm, they manifested broad social knowledge despite their perceptional and cognitive immaturity. Why did infants demonstrate such different reactions in two different research paradigms?

As noted above, the difference between the two research modes is that the “Observer” procedure only observes infants’ behavior in familiar, comfortable conditions. In contrast, the “Experimenter” procedure created unfamiliar or unusual stimuli for participants: unfamiliar tasks, in a new environment, with a new person, etc. Studies on emotion contagion and memorizing in learning show increased cortisol levels of participants during a public performance (e.g., Haley et al. 2006; Brand et al. 2018). It appears obvious that unusual situations and unfamiliar tasks during experiments within the “Experimenter” procedure could emotionally stimulate participants. It seems that the above-noted emotional stimulation in dyads during experiments under the “Experimenter” paradigm could create an extended property of newborns’ perception through an emotional connection with adults. The emotional bond evokes intentionality in neonates. Indeed, infants were not alone during decision-making process in the experiments under the “Experimenter” paradigm. Therefore, it is not too controversial to say that young children solved these complex tasks not independently while being together with their mothers and on rare occasions with other caregivers. Experimental data also show that preferences of infants can depend on their caregivers (Quinn et al. 2002; Bar-Haim et al. 2006; Gaither et al. 2012). 

Intentionality and intention are separate concepts as these bear different semantics and reflex different appearances. According to the accepted view, the intention is a conscious manifestation, i.e., a prior conscious decision to conduct a behavior (Danilov and Mihailova 2021c). Unlike intention, intentionality may manifest without the desire and awareness of the individual, or unconsciously as well. For instance, people could be unable to stop thinking regarding a traumatic life event and would think obsessively regarding the event to the point of distraction and an inability to function in their daily life (Bargh 1990). At least partly, the difference between intentionality and intention manifests as shared intentionality (Danilov and Mihailova 2021c). It is clear that shared intentionality in caregiver-infant dyads depends on both the caregiver’s intentionality and an infant’s ability to be intentional towards a phenomenon. The current paper supposes that, in experiments, children could obtain a caregiver’s help, solving tasks through shared intentionality. This analysis shows that the period of an appearance of intentionality in infants depends more on social interaction features than on child’s age.

After Vygotsky we know that infants’ cognition develops in social interaction. Interactions with others lead to internalization of cognitive processes first achieved in the social context (Vygotsky 2016). Could the research mode of testing newborns in the presence of their mothers (or caregivers) reveal pure newborns’ responses on stimuli? It seems not too controversial to stay that experiments should consider adult-child emotional bonds when evaluating children’s performance. The opposite opinion may retort that even for protoconversation, what should the medium for cues be, considering that interaction with newborns through sensory cues is impossible? This other view may continue reasoning with an idea of inherited knowledge and/or specific brain structures (or embodied dynamic system) that separately or together launch cognition by grasping only information patterns relevant to them. Adepts of these two opposing approaches are finding consensus in future research. However, empirical data already permit developing a digital method for assessing cognition in children through measuring shared intentionality, even if the essence of such interaction is still undefined. 

In addition to the above-noted challenges, two limitations of this new paradigm for assessing cognition in children through measuring shared intentionality can be noted here. These limitations are crucial in determining the dyads’ features that ensure protoconversation.

### 5.1. Limitation 1: Shared Intentionality Appears Not in All Interpersonal Dynamics

Shared intentionality is the central point of protoconversation (Tomasello 2019). However, this specific method of interaction is probably not present in all interpersonal dynamics. Can each caregiver efficiently contribute to this testing, or only one who shares an everyday routine with the child? In the middle of the 20th century, the discussion on inherited circadian oscillators introduced a concept of entrainment which provides a timing cue for a biological rhythm (APA Dictionary n.d.). It is a biochemical oscillator that cycles with a stable phase and is synchronized with solar time. For example, the production of gonadal hormones in seasonally breeding animals can be a result of entrainment to increasing day length (APA Dictionary n.d.). In the study of chronobiology, entrainment occurs when rhythmic physiological or behavioral events match their period to that of an environmental oscillation (Olds 2015). Ultimately it is an interaction between circadian rhythms and the environment. Exposure to certain environmental stimuli will cue a phase shift, and an abrupt change in the timing of the rhythm. This rhythm persists even in the absence of environmental cues because it is not a learned behavior but something that is inherent in organisms (Olds 2015). Entrainment helps organisms maintain an adaptive phase relationship with the environment as well as prevent drifting of a free running rhythm. This stable phase relationship achieved is thought to be the main function of entrainment (Olds 2015). According to Semin and Cacioppo (2008), the movements of other human beings can be mapped isomorphically onto our own bodies. This means that the infants’ adaptive phase relationship with the environment (together with their mother) helps in capturing her movements.

This notion was later also applied to humans’ capacity to become entrained with one another or with an external stimulus: ‘the synchronization of blind mice, which were previously free running, following the addition of normal seeing mice under cyclic light-dark conditions, suggests that social Zeitgebers exist (Aschoff 1963, p. 588)’. ‘The term “zeitgeber”, German for “time-giver”, is used to describe environmental, or external, time cues that entrain human circadian rhythms (Grandin et al. 2006).’ According to Grandin et al. (2006), the social rhythm metric is intended to capture an individual’s “social rhythm”, or the frequency with which daily activities are performed and the level of regularity and social contact associated with these activities. The timing of routine activities (i.e., waking up, eating meals etc.) constitutes the social rhythm of individuals (Grandin et al. 2006), and these bricks of everyday life are social zeitgebers that create coordinated interpersonal dynamics between persons involved in the encounters within this social rhythm. The goal of synchronization in circadian systems is not simply “synchrony”—i.e., equal “speed” in the two systems—but “phase control”: a clearly defined and stable phase–angle difference between the biological oscillation and the Zeitgeber (Aschoff 1963).’ Given the above arguments, the paramount quality of this notion is acceptance by all participants (on the biological level) of the social zeitgeber rhythms (cyclical routine stimuli, i.e., waking up, mealtimes, exercise, clocks) which influence individuals in a large time scale, affecting their life during months or even years. These soft, while cyclical, routine stimuli are formed by both rhythmical changes of natural environment stimuli and cyclical social stimuli of individuals involved in the encounters within a certain social rhythm. Social entrainment promotes shared biological rhythm in individuals–physiological coordination of each other. However, as the MCI supposes, Coherent Intelligence requires psychophysiological coherence in individuals, which is the first stage of the interpersonal dynamics uplift. Supranormal stimuli are essential for emotional stimulation to shape psychophysiological coherence further. Only the combination of physiological dependence with emotional arousal leads to participants’ psychophysiological coherence. The following subsection reflects the role of emotional arousal and contagion in the psychophysiological coherence of individuals and, consequently, group performances. For example, in caregiver-infant dyads, only the caregiver who shares the everyday routine with the child can contribute to detecting the child’s developmental delay in computer testing if they are emotionally stimulated.

### 5.2. Limitation 2: Shared Intentionality Appears Not Always at the Same Degree

Protoconversation through shared intentionality is probably not maintained by dyads at the same degree of elevation all the time. Does the distance assessment procedure guarantee emotional stimulation during testing? As mentioned above, empirical evidence shows increased cortisol levels of participants during a public performance (e.g., Haley et al. 2006; Brand et al. 2018) that refers to emotional arousal. Therefore, online computerized testing can also provide emotional stimulation under certain conditions. However, a degree of emotional arousal is also crucial for shared intentionality. Elevated cortisol levels impair cognitive processes. Empirical evidence supports the law of Yerkes and Dodson (1908) that optimal but not maximal arousal predicted the highest performance (e.g., Henckens et al. 2011, 2012; Quesada et al. 2012; Schwabe et al. 2013; de Veld et al. 2014; Brand et al. 2018).

### 5.3. Limitation of the Study: A Lack of Knowledge about Physical Processes

The limitations of the current study appear from a lack of knowledge about physical processes during social interaction. Indeed, understanding the crucial conditions (in specific, the physical processes) during an experiment determines the requirements for and imposes restrictions on the dependent and independent variables of the experiment. The correct definition of the circumstances influencing the expected results is essential for establishing the main empirical question that should be investigated. At this point, social sciences should meet physics. Piaget argued that physics is not yet a truly general science since it remains artificially limited to the study of inert matter. This eminent psychologist claimed that complete physics would also study living beings, especially thinking ones. Even though the notion of protoconversation is generally accepted and used in social science to illustrate interaction in the mother-child dyads, its essence is still undefined from physics. Social interaction modality appearing at the beginning of cognition challenges the design of experiments in many social science disciplines and their previously obtained results.

On the other hand, it shows a new possible framework for developing the quality, reliability, and value of experiments’ results for more responsible research in social science. This innovative approach can develop novel tools to prevent misconduct and safeguard validity and reliability in research. A biological and/or physical basis of Shared Intentionality is a challenge that can contribute to understanding cognition and developing research quality.

### 5.4. Implication: Indicating Development Trajectory 

Does the digital assessment of shared intentionality in a dyad detect developmental trajectory in young children? Can online computer-based testing of interaction skills detect a developmental delay in children younger than the age when the typical trajectory of peers’ development determines verbal communication?

(1) A deficit in basic communication and social interaction skills can indicate developmental delay (Gresham and McMillan 1997; Cullinan 2002; Prior and Ozonoff 2007; Pierce et al. 2019; Rojas-Torres et al. 2020). According to Tomasello et al. (2005), great apes (and some children with autism) understand the basics of intentional action, but they still do not participate in activities involving joint intentions and attention—ShI. The lack of ShI leads to a developmental delay in children, following the previous argument.

(2) Shared intentionality is the primal tool of protoconversation between young children and caregivers. It appears in infants before communication does. The studies mentioned above show that stimulation of ShI in dyads can facilitate children’s cognition, but on the other hand, the lack of ShI leads to developmental disability. For this reason, the detection of its deficiency can indicate a deviation in the trajectory of development. A ShI assessment shows the trajectory of communicative skills development in infants at the earliest stages before explicit markers of communicative skills appear.

(3) According to Danilov and Mihailova (2020), the interpersonal dynamics of individuals in psychophysiological coherence promote coordinated neuronal processes in humans and are defined as the Model of CI. Ever-increasing coordination of movements (from imitation to interactional synchrony) overlaps ever-growing emotional experience (from arousal to contagion) (see Figure 1, Danilov and Mihailova 2020). Finally, ShI in the dyads provides a categorization of reality promoting CI—the children’s distinct perception of sudden insights in knowing new phenomena. The Model of CI yields the particular structure of emotion-motion interpersonal dynamics that is valuable to predictable stimulation of efficient group collaboration and an assessment of a CI extent. 

Given arguments (1 + 2 + 3) together, measuring the extent of ShI in dyads can indicate the developmental trajectory of children. Suppose computer testing can measure the interaction ability in infants before the age when the typical developmental trajectory predetermines verbal communication (about in 18–24 months of age and rare cases up to 36 months). In that case, a deviated outcome can mean an alert (comparing with normal trajectory baseline). 

At the same time, it is well known that the traditional paradigm of assessing through behavioral markers in children has several difficulties. Zablotsky et al. (2019) reported that about 17% of children aged 3–17 years were diagnosed with developmental disabilities during a study period of 2009–2017 (e.g., 9.04% children with attention-deficit/hyperactivity disorder (ADHD), 7.74% with learning disability (LD), 1.10% with intellectual disability, and 1.74% children with Autism Spectrum Disorders (ASD)). One of the crucial issues in diagnosing and treating many types of developmental delays in children is efficient markers at the earliest stage (Pierce et al. 2019; Zablotsky et al. 2019; McCarty and Frye 2020; Rojas-Torres et al. 2020). The earliest possible assessment of a developmental delay provides early intervention. 

A growing body of literature presents a cohort of detecting autism methods in young children based on assessing behavioral markers (Barbaro and Yaari 2020; Dahiya et al. 2020; Rahman et al. 2020). The only proven therapy for core symptoms of ASD is behavioral therapy (McCarty and Frye 2020). The earlier an intervention is started, the better the long-term outcomes for the child (Pierce et al. 2019; Hyman et al. 2020; McCarty and Frye 2020). However, the vital issue with ASD diagnosis (through behavioral markers) is performing efficient feature selection before implementing classification; efficient feature selection can support accurate and effective classification (Rahman et al. 2020). However, several studies have shown that early signs of ASD can sometimes be detected using parent report screens as early as 12 (Wetherby et al. 2008; Turner-Brown et al. 2013; Pierce et al. 2011) or 18 months of age (Chlebowski et al. 2013; Robins et al. 2014), ASD diagnostic certainty may come only around age 3 (Zwaigenbaum et al. 2009; Volkmar et al. 2014; Baio et al. 2018; Pierce et al. 2019). This diagnostic lag is because, first, although the social communication features of ASD are present before that time, frequently some of them are not yet fully present (Volkmar and Wiesner 2009). Second, difficulties in early diagnosis reflect the intrinsic problems in the assessment of very young children (Volkmar et al. 2014). Third, there is the potential for rapid developmental changes even without intervention (Volkmar et al. 2014)—ongoing development yields an extent of uncertainty in developmental diagnosis. Fourth, the behavior markers in diagnosing bear limitations that require parents’ competence in reporting and the level of experience of the professionals. The cohort of different methods of ASD diagnosis is settled into four main subsets: Psychological Assessments, Speech-Communication Assessments, Medical Assessments, and Occupational and Physical Therapy Assessments (Volkmar et al. 2014). However, the level of experience of the professionals involved in the assessment is essential for diagnosis (Volkmar et al. 2014) since behavioral markers used as the primary tool in many methods depend on their interpretation in a particular context. Despite advanced knowledge of the genetic basis of autism, even the medical assessment subset of the methods does not yet provide a simple laboratory test for autism (Volkmar et al. 2014). There has still been a deficiency in classification performance; methods based on behavior markers require further research for the accurate and efficient classification of ASD (Rahman et al. 2020). These above circumstances limit the development of digital computerized testing for the early detection of ASD based on behavior markers.

Attention-deficit/hyperactivity disorder (ADHD) is a debilitating mental health disorder most frequently diagnosed in school years (Sonuga-Barke et al. 2011). ADHD is marked by symptoms of inattention, overactivity, and impulsiveness that have an early onset. They are age-inappropriate, persistent, and pervasive (Swanson et al. 1998; Sonuga-Barke et al. 2011). However, these markers are not universal. Longitudinal studies demonstrate that high levels of preschool ADHD symptoms do not always persist into the school years and later life (Campbell et al. 1994; Lavigne et al. 1998; Mathiesen and Sanson 2000). Many studies have focused on such behavior problems among children from 3 years of age. Even though behavior problems at 18 months are relatively common, we can begin to predict which children will have persisting problems after the early childhood phase (Mathiesen and Sanson 2000).

Learning disabilities are developmental disorders that usually manifest during education at school (Kulkarni et al. 2001). Behavioral markers are only available for determining LD in children (Stock et al. 2007). Skills to be assessed are (i) procedural counting knowledge, (ii) the ability to seriate in preschool, and (iii) the conceptual counting knowledge of young children). In particular, the inability to do such things in preschool (at the age 5 to 6) may be a marker for later arithmetic disabilities (Stock et al. 2007). Further research requires finding an accurate and efficient behavior classification since young students with LD also manifest behavior problems and interaction difficulties (Cullinan 2002; Gresham and McMillan 1997).

Some developmental delays have an identifiable cause; for others, the delay’s cause is not clear. However, many possess a common feature-children’s lack of interaction ability (Erikson 1963; Winnicott 1973; Prior and Ozonoff 2007; Sternberg and Sternberg 2012; Vygotsky 2016; Pierce et al. 2019; Rojas-Torres et al. 2020). It is common for children with developmental delays to have difficulty with social and emotional skills. Their diagnosis consists of a behavioral assessment paradigm, which requires improving accuracy, accessibility, and efficiency.

Digital computer-based testing of interaction skills (shared intentionality in the absence of sensory cues) can overpass the above-noted difficulties, assessing non-verbal children without applying the procedure with behavioral markers. This digital method for assessing cognition can detect a developmental delay in children younger than the age when the typical trajectory of peers’ development implies verbal communication. This assessment method by Danilov et al. (2021a) could detect the interaction ability of even an 18-month-old non-speaking child. The testing protocol of the experiments with infants by Danilov et al. (2021a) could become the framework for further research for developing an online computer-based method of developmental delay assessment of toddlers aged 18 to 36 months. This article proposes a new assessment paradigm for earlier detection of developmental delay, different from assessing through behavior markers. Its fast, simple, and practical use can make this tool a resource for early detection of “alarm bells” in children’s cognitive development to the widest possible audience.

The crucial issue of this new paradigm is a design for how this assessment tool can evaluate protoconversation in dyads. We believe that it is based on protoconversation testing when children are asked to answer unfamiliar and unintelligible tasks (familiar and intelligible to caregivers). For instance, recent research showed detection of this interaction modality through the block of 10 tasks to choose the proper set of items (from 1 to 3 balls) that the experimenter asked in an unfamiliar language (Danilov et al. 2021a, 2021b). Children with typical developmental trajectories showed 2–3 times better interaction with their mothers (2–3 times more correct answers) than the child with developmental delay (see Section 4). The current article aims to create a conceptual framework for future research on digital methods for assessing cognition in children through estimating shared intentionality. Further research should answer the following questions: (a) what is an average (a medium) magnitude of the protoconversation in children with the typical developmental trajectory? This average becomes the baseline for assessing developmental delay in children because a results’ deviation means possible developmental delay. (b) Does the certain magnitude of deviation from average protoconversation in children relate to the specific developmental impairment? Alternatively, does the reduced magnitude of protoconversation only show a developmental delay in general, without any association with specific impairment—causing a developmental delay?

## 6. Conclusions

This paper proposed the concept design for further research to develop the digital assessment tool for unbiased detecting (even online) developmental delay in children at the earliest stage from 18 months of age. It poses a new assessment paradigm for earlier detection of developmental delays, different from assessing through behavioral markers. This method assesses protoconversation outcomes in dyads by analyzing meaningful (comprehended) interaction results. Measuring protoconversation outcomes relies on a recent study on protoconversation outcomes in infants and toddlers. Danilov et al. (2021a) tested shared intentionality in dyads with young children belonging on standard developmental trajectory (18 months, 28 months, 31 months of age), comparing their results with the child with a developmental delay (aged 33 months). Their 7 min online computer-based assessment testing verified children’s ability to acquire knowledge through shared intentionality showed three times lower results of children with developmental delays. 

This conceptual design discussed the twofold research problem. First of all, it discussed unexpected achievements of infants, observing the literature about children’s development. Two experimental modes for observing behavior in infants were inferred. The analysis supposed that due to the caregiver’s help under emotional arousal, newborns’ intentionality could appear even before children’s intention occurs. The article argued that social entrainment and emotion-motion interpersonal dynamics shape shared intentionality. Therefore, newborns manifested unexpected achievements (e.g., solving unintelligible tasks) by performing them together with caregivers. 

The current study hypothesized that if shared intentionality promotes cognition from the onset, this interaction modality can also facilitate cognition in older children. Therefore, in the second step, the study develops knowledge towards a digital assessment that can indicate the extent of children’s ability to acquire knowledge through protoconversation. This specific assessment can detect a lack of communication ability in children. Since the latter is one of the circumstances that cause a developmental delay, this test can be an efficient digital tool for understanding cognitive development trajectories in children. This computer-based testing of protoconversation can detect a developmental delay in children younger than the age when the typical trajectory of peers’ development implies verbal communication. Further research should determine their contribution to the protoconversation in dyads and the degree of infants’ ability to acquire knowledge through social interaction. The proposed testing protocol (following the research method presented in Section Materials and Methods by Danilov et al. 2021a) could become the conceptual framework for further research to develop an online computer-based method of developmental delay assessment for children aged 18 to 36 months. Further research can validate the 7–10 min computerized quiz (designed following the method by Danilov et al. 2021a). This article proposed a theoretical framework for future research on digital method for measuring shared intentionality.
